# Impact of differences in clinical training methods on generic skills development of nursing students: A text mining analysis study

**DOI:** 10.1016/j.heliyon.2019.e01285

**Published:** 2019-03-14

**Authors:** Hyeyong Lee, Rie Shimotakahara, Akimi Fukada, Sumiko Shinbashi, Shigemitsu Ogata

**Affiliations:** a Department of Fundamental and Clinical Nursing, School of Health Science, Faculty of Medicine, Kagoshima University, 8-35-1 Sakuragaoka, Kagoshima, 890-8544, Japan; b Faculty of Neurology Gross Anatomy Section, Kagoshima University Graduate School Medical and Dental Sciences, 8-35-1 Sakuragaoka, Kagoshima, 890-8544, Japan; c Independent School for Integrative Medical Specialist in Kanazawa, 7-1 Horikawasinnmachi, Kanazawa, Ishikawa, 920-0849, Japan

**Keywords:** Health profession, Education

## Abstract

**Background:**

Discussions and investigations are being conducted in regard to the question of how, instead of acquiring specific kinds of expertise at university, students can instead be taught “generic skills,” which are the competencies for engaging in the everyday life of a working adult.

**Objectives:**

This survey was aimed at assessing the impact of clinical practical training on generic skills from the perspective of student's own perceptions of personal growth. And we compared different three nursing colleges of the practical training methods to investigate the associations between generic skills and practical training methods.

**Design:**

A text mining analysis study.

**Settings and participants:**

The study was conducted with the participation of Japanese third-year students who had completed clinical practical training at three nursing colleges. Study period was December 2016 to February 2017. There were 242 participants in total, and valid responses were obtained from 216 participants.

**Methods:**

We distributed an anonymous self-administered questionnaire. Data collected from open-ended responses was subjected to analysis using text mining methods.

**Results:**

A total of 2,903 words were extracted as the result of analyzing text data for a total of 568 sentences. From the patterns in which the extracted words appeared, we were able to classify details reported by students about the areas where they recognized personal growth into nine categories. We found “teacher,” “now,” and “clinical instructor” among the characteristic words for School A, as well as “learn,” “records,” and “planning.” The terms extracted for School B included “acquired,” “communication,” and “consideration.” Terms extracted for School C included “perform,” “human,” and “action + can.”

**Conclusion:**

Students perceive more growth in terms of generic skill competencies than in terms of expert knowledge or techniques. Project-based learning is associated with students' “ability to discover problems,” while experience-based practical training is associated with students' “ability to sustain action.”

## 1 Introduction

Our contemporary society of advanced globalization and information technology requires flexible thinking skills and the ability to deal with change (Spencer and Spencer, 1993; Rychen and Salganik, 2003; Yoshihara, 2007; Shimizu, 2012; Griffin and Care, 2015; Kuroda and Kumano, 2018; Martin, 2018). Accordingly, discussions and investigations are being conducted in regard to the question of how, instead of acquiring specific kinds of expertise at university, students can instead be taught “generic skills,” which are the competencies for engaging in the everyday life of a working adult (Shimizu, 2012; Kubota, 2013; Griffin and Care, 2015; Yasuda et al., 2016; Kinoshita, 2017; Balderas et al., 2018).

Generic skills, which received increasing attention in the West from the 1980s through the 1990s, are now also known as “employability skills” and “transferable skills” in connection with career education (Kinoshita, 2017). In line with this trend, the Japanese Ministry of Economy, Trade and Industry (METI) has set forth guidelines for training in “Fundamental Competencies for Working Persons,” while the Central Education Council has defined and reported findings on “academic abilities.” In addition, employment training projects led by the Japanese Ministry of Education, Culture, Sports, Science and Technology (MEXT) explicitly refer to the cultivation of “generic skills” through university education (Kubota, 2013). Although expressed in various ways, each of these terms indicates those competencies required for any kind of work in society and defined as“generic skills” in this study. That is, they refer not to specific vocational knowledge and skills, but to fundamental qualifications and abilities required for any occupation, and which are situated between basic education and the specialized education. In recent years, this is the chief aim of university education to develop generic skills (Kubota, 2013; Griffin and Care, 2015; Kinoshita, 2017). Key competencies are (1) logical thinking skills, (2) critical analysis skills, (3) the ability to form interpersonal relationships, and (4) the ability to act independently (Ministry of Education, Culture, Sports, Science & Technology in Japan, 2010; Cabinet office Government of Japan, 2014).

As fostering generic skills has been gaining worldwide attention, the concept of “literacy” used by the OECD's Programme for International Student Assessment has had a particularly significant impact, as have the “key competencies” similarly proposed by the OECD's DeSeCo (Definition and Selection of Competencies) Project (OECD, 2004; OECD, 2005). There have been some attempts at developing methods for quantitatively assessing generic skills, such as the OECD's Programme for the International Assessment of Adult Competencies (OECD, 2013) and its Assessment of Higher Education Learning Outcomes program (OECD, 2012), but in the Japanese context, trial efforts at such quantitative assessment are only now beginning, including the Progress Report on Generic Skills used in this study (Kawaijuku Educational Institution and RIASEC, Inc., 2015).

The cultivation of these skills is also an urgent challenge for basic nursing education, necessitating the search for ways to train students who are able to solve their own problems and apply things they have learned (Ministry of Health, Labor and Welfare in Japan, 2011; Korean Accreditation Board of Nursing Education, 2012; Unesawa et al., 2012; Choi and Kim, 2016). However, it has been difficult to achieve this because recent nursing students tend to be socially immature because of their limited life experiences, insufficient mastery of social norms, and poor communication skills (Ministry of Health, Labor and Welfare in Japan, 2011). This is a common challenge in nursing education settings even outside Japan. Rather than learning theory-centered knowledge, it is of the utmost importance to cultivate the ability to seek solutions and to be proactive in thinking, acting, and improving upon the results (Park and Bae, 2008; Yang and Moon, 2007).

Nurses must continue learning on a lifelong basis (Park and Bae, 2008; Hattori et al., 2015). In particular, clinical training is an important educational setting for building practical nursing competencies while actually engaging with patients, nurses, and other medical staff (Waguri, 2010; Ministry of Health, Labor and Welfare in Japan, 2011; Ministry of Education, Culture, Sports, Science & Technology in Japan., 2011). Moreover, while it can be inferred that clinical training has an impact on not only practical nursing competencies but also nursing students' generic skills, to the best of our knowledge, no studies of this effect have yet been reported.

Accordingly, this survey was aimed at assessing the impact of clinical training on generic skills from the perspective of student's own perceptions of personal growth. And we compared different three nursing colleges of the practical training methods to investigate the associations between generic skills and practical training methods.

## 2 Methods

### 2.1 Study design

This survey was cross-sectional exploratory study to examine the effect of clinical training on generic skills from the perspective of students' perceived self-growth. And, it was mixed methods research using the text mining analysis.

### 2.2 Study setting

#### 2.2.1 Participants and background

The study was conducted with the participation of Japanese third-year students who had completed clinical training at three nursing colleges. Nurse training courses consist of the following five fields: Basic Fields, Specialized Basic Fields, Specialized Fields I and II, and Integrated Fields in Japan. These are set out in the Regulations of the Educational Institutions of Public Health Nurses, Midwives, and Nurses, and nursing education is implemented at individual nursing training centers based on the number of academic credits and the educational content of the Designated Regulations (Act on Public Health Nurses, Midwives, and Nurses, 1948). The present study was conducted with the participation of third-year students who had completed all clinical training in the five areas defined in Specialized Fields II.

The background and characteristics of the training method of each school are shown in Table 1. Since the clinical training at School A involved project-based learning, students themselves reflected on goals and tasks using practical learning portfolios. At School B, students carried out recordings and evaluations based on the clinical course of patients in their care. At School C, experience-based practical training was introduced involving students caring for multiple patients together with nursing staff, with students discovering the meaning of nursing by reflecting on nursing practice.

**Table 1:** Background and characteristics of the training method of each school.

	School A	School B	School C
Number of credits	16	16	16
Period	from June to January	from September to February	from August to February
Number of students	92	84	66
Clinical training methods	Project-based Learning	Methods focused on the development of the nursing process	Experiential learning
	• Use a “vision goal sheet” and “reflection sheet” for clinical training. The “vision goal sheet” is a goal attainment sheet intended to eliminate deviations from intended goals by having students fill in the sheet before starting practical training and maintain learning motivation by checking the goals from time to time during their practical training. On the “reflection sheet,” students compose reflections in accordance with their individual skills, namely “reflections” and “self-awareness.”	• Develop nursing process by organizing and analyzing information about patients' health problems and then implementing and evaluating nursing plans. • Verify the assistance that patients require based on nursing theory.	• This is a method that involves turning events encountered in day-to-day practical nursing training into learning opportunities and taking them as a process by which students search for their own solutions to the problems in question. In this way, students' individual nursing experiences and students' subjectivities are placed at the core of the practical training process. Teaching staff elicit students' lived experiences and support the experiential learning entailed by the process of reflecting upon and conceptualizing these experiences and then making use of them in subsequent practice. • In experience-based practical training, to think and act like a student, individual students act in line with nurses and provide nursing care to patients cared for by the nurse.
Records	Vision-goal sheets Reflection notes Relationship diagram	Face sheets Nursing diagnosis Relationship diagram Nursing care plan Progress notes	Nursing diagnosis Relationship diagram Nursing care plan Progress notes Reflection notes
Goal	• The aim is for students to discover their own problems for themselves. Visualizing one's own achievements, sharing them with others, and evaluating them allows clarification of the course of the reflection cycle of Description→ Feelings→ Evaluation→ Analysis, which can subsequently lead to Conclusion → Action Plan. • The above also applies to subject-oriented nursing and can offer solutions to the problems that patients face.	• Through the integration of acquired knowledge and skills in nursing, cultivate practical nursing skills for being able to clarify nursing-related problems experienced by patients and provide nursing assistance needed to address the problems. • Cultivate the ability to think of patients as living persons and design nursing assistance from a long-term perspective.	• Clarify nursing problems and to be able to provide the nursing assistance required to solve problems being faced by patients and their families. • Master the ability to be able to make decisions and act with initiative and cultivate a foundation for lifelong learning. • Learn supplementary techniques for various nursing experiences and medical treatments under the guidance of veteran nurses.
Clinical training area	Adult （6） Gerontological （4） Pediatric （2） Maternal （2） Phychiatric （2）	Adult （6） Gerontological（4） Pediatric （2） Maternal （2） Phychiatric （2）	Adult （6） Gerontological（4） Pediatric （2） Maternal （2） Phychiatric （2）
Training facilities	Various hospitals (independent school)	University hospital (school attached to university hospital)	Various hospitals (independent school)

Notes: Since the clinical training at School A involved project-based learning, students themselves reflected on goals and tasks using practical learning portfolios. At School B, students carried out recordings and evaluations based on the clinical course of patients in their care. At School C, experience-based practical training was introduced involving students caring for multiple patients together with nursing staff, with students discovering the meaning of nursing by reflecting on nursing practice.

#### 2.2.2 Definition of terms

Project-based learning: This method of learning involves students, rather than being assigned challenges, discovering challenges for themselves and working to solve them to realize their vision. This involved the use of a Vision/Goal Sheet (what students want to accomplish, and why) and a Reflection Sheet (for introspective contemplation) (Suzuki, 2010).

Experience-based practical training: This method of learning involves students discover meaning for themselves through their experiences of complex phenomena. It is a learning method in which students learn from repeated introspective experiences through their direct involvement with patients, their families, and medical staff (Akutagawa and Katsuyama, 2007).

#### 2.2.3 Study period

December 2016 to February 2017.

#### 2.2.4 The procedures of conducting

After providing participating students with an oral and written explanations of the purpose and methods of the study and obtaining their consent, we distributed an anonymous self-administered questionnaire within two weeks after their practical training. The survey was implemented using the placement method, with students depositing completed surveys into a collection box within a week. And those questionnaires were collected by us.

### 2.3 Contents of questionnaire

#### 2.3.1 Basic information of participants

Responses were obtained regarding participants' sex, age, level of educational attainment, and employment experience.

#### 2.3.2 Open-ended questionnaire items

Descriptive responses were obtained to the question “In which areas do you think you have developed through clinical training?” In this study, “development” was defined as “self-growth as perceived by the students themselves.” Students were asked to provide open-ended responses about their “development,” which then served as the basis for the analysis of internal development.

### 2.4 Data analysis method

#### 2.4.1 Data preparation

Data collected from open-ended responses was subjected to analysis using text mining methods. The following analyses was conducted using Text Mining Studio 6.0.3 (NTT DATA Mathematical Systems Inc.) and KH coder (ver. 2).

In procedural terms, text mining involved first digitizing the collected textual data. Next, morphological and syntactic analyses were carried out to enable the computerized processing of the textual data. Morphological analysis entails dividing strings of text into grammatically meaningful component units and determining their respective grammatical features (e.g., parts of speech) (Berry, 2004; Sukanya and Biruntha, 2012). Parsing analysis shows the grammatical and semantic relationships between components to find the dependent relationships between them (Ozao and Ishida, 2009). New qualitative data were obtained by performing these conceptual extractions.

#### 2.4.2 Hierarchical cluster analysis

Hierarchical cluster analysis (Ward's method, Jaccard coefficient 1.0) was conducted with regard to areas of growth recognized by the students themselves. Hierarchical cluster analysis is an analytical method that captures differences in the properties of each data as “distance” and expresses similarity in terms of the magnitude of the distance (Higuchi, 2004). After carrying out morphological analysis using KH Coder and digitizing the textual information in the sentences, we searched for word combinations with similar patterns featuring the extracted words. In this analysis, from the tree diagram that was generated as the result, we confirmed one by one the formation of possible clusters by a process of classification. Results were classified after comparison with concepts of competency formation for generic skills as reported in the literature.

#### 2.4.3 Correspondence analysis

Taking the three participating schools as attributes, a correspondence analysis was carried out in order to determine whether there was any relation between the schools and the extracted words/phrases. The correspondence analysis, which was carried out as part of the text mining analysis, shows the distribution of attributes as mediated by words/phrases by mapping the distribution of relationships between words/phrases and expressions and their attributes in two-dimensional space (Sukanya and Biruntha, 2012). Attributes that have words/phrases and expressions used in a similar manner were positioned in close proximity to each other, and their relationships were analyzed based on their respective distance.

#### 2.4.4 Characteristic word analysis

In characteristic word analysis, the objects of analysis were separated by school, whereupon characteristic words were extracted based on consideration of the frequencies of individual words based on the degree of complementary similarity as an index. Characteristic words are terms whose appearance is measured not in terms of simple frequency, but according to their bias toward certain attributes after the distribution of terms is considered. This study used complementary similarity as an index value based on the notion that words appearing more frequently are more characteristic of the attribute (Ozao and Ishida, 2009). The higher the number of occurrences of a given word in the analysis, the lower the probability that the word associated with the attribute coincidentally, enabling the word to be regarded as more characteristic. A higher index value indicates that a term is more characteristic of the attribute in question.

#### 2.4.5 Qualitative analysis

A function referring to the original text was used to bring added depth to our consideration of the words/phrases extracted from these three analyses. After checking the context of the original descriptive data, we examined the nature of the competency. As a part of this process, five researchers with previous experience in text mining analysis repeatedly checked the original text and worked to ensure its validity by confirming and verifying whether their interpretations were influenced by any preconceptions.

Because this study analyzed descriptive data in Japanese, it was felt that the results of an analysis based on Japanese grammar would be more faithful to the data. When writing up the results of the Japanese analysis and converting these to English, we referred to previous studies of nursing pedagogy, investigating them based on the words and phrases used. We also referred to the Igaku Chuo Zasshi (ICHUSHI) thesaurus and glossary and checked corresponding terms in English using Medical Subject Headings (MeSH^®^) terminology. Further, the validity of the translation was assessed not only by researchers but also with the assistance of native English-speaking checkers.

### 2.5 Ethical considerations

The survey was conducted using an anonymous questionnaire. It was implemented after obtaining approval from the ethics review committee of our school (Approval No.290).

After obtaining the approval of the ethics committee, we provided participating students with an oral explanation of the purpose of the study and its methods, specifying that the survey was anonymous and personal information would not be published, that their participation in the study was voluntary, that they would not be disadvantaged in any way should they choose not to participate, that the resulting data would not be used for any purpose other than the survey, and that they were free to withdraw after choosing to participate, and that their anonymity would be maintained. The return of the questionnaire by posting to the collection box was regarded as an indication of participants' consent.

## 3 Results

There were 242 participants in total, and valid responses were obtained from 216 participants (valid response rate: 89.26%). The breakdown was as follows: School A, 81 participants (37.50%); School B, 76 participants (35.19%); and School C, 59 participants (27.31%). There were 29 men (13.43%) and 187 women (86.57%), with participants aged between 20 and 29 years old accounting for approximately 90% of the total. These included 24 students (16.2%) who had enrolled after graduating from vocational schools or universities, and 32 (11.1%) who had previous employment experience.

### 3.1 Analysis of open-ended responses concerning “self-growth”

A total of 2,903 words were extracted as the result of analyzing text data for a total of 568 sentences. Broken down by part of speech, these consisted of 1,703 nouns (58.66%), 803 verbs (27.66%), 183 adjectives and nominal adjectives (6.3%), and 214 other words (7.38%). The nouns, verbs, adjectives, and nominal adjectives, which accounted for over 90% of the total were selected for analysis. The rest of the words, which included mainly particles, adverbs, and symbols were excluded from the analysis, as these lacked any semantic content of their own. From the viewpoint of protecting personal information, proper nouns such as personal names and the names of organizations were also excluded from the analysis, as were numerals and pronouns, which would be meaningless in the analysis. This left a total of 2,624 words for analysis.

#### 3.1.1 Hierarchical cluster analysis

A total of 46 words were extracted, which were then able to be categorized into 9 clusters from the strength of their mutual relationships (Table 2). From the constituent words and original text comprising these clusters, the contents of these clusters were summarized as follows: the cluster made up of the words “surroundings/myself/behavior/think/adjustment/plan” was regarded as “practical skill”—namely, “the ability to put actions into practice in line with effective planning.”

**Table 2:** Cluster analysis of self-growth (n = 855).

**Cluster 1: Practical Skill**		**Cluster 6: Planning skills**	

**Term**	**n**	**Term**	**n**

Surroundings	17	Knowledge	15
Myself	83	Need	37
Behavior	39	Patient	42
Think	44	Nursing	35
Adjustment	17	Recognize	21
Plan	11	Have	17
		Investigate	12

**Cluster 2: Collaborative Ability**		**Cluster 7: Creative confidence**	

**Term**	**n**	**Term**	**n**

Supervise	13	Learn	12
Consultation	14	Seem	12
Report	12	Study	27
		Pleasant	13

**Cluster 3: Affinity**		**Cluster 8: Ability to discover problems**	

**Term**	**n**	**Term**	**n**

See	20	Learning	4
Human	17	Growth	15
Understanding	10	Information	5
Other people	13		
Communication	35		
Involve	12		
Feel	11		

**Cluster 4: Ability to sustain action**		**Cluster 9: Emotional control ability**	

**Term**	**n**	**Term**	**n**

Ability	35	Member	10
Practice	17	Increase	19
Positive	22	Speak	15
Know	20	Practical training	79
		Can	55
		Talking	20
		Friend	20
		Teacher	18

**Cluster 5: Leadership**			

**Term**	**n**		

Opinion	20		
Say	12		
Public	7		
Speak	4		

Notes: Hierarchical cluster analysis is an analytical method that captures differences in the properties of each data as “distance” and expresses similarity in terms of the magnitude of the distance. A total of 46 words were extracted, which were then able to be categorized into 9 clusters from the strength of their mutual relationships.

Similarly, from the configuration of the words shown in Table 2, the remaining 8 clusters were named “collaborative ability,” “affinity,” “ability to sustain action,” “Leadership,” “planning skills,” “creative confidence,” “ability to discover problems,” and “emotional control ability.” All of 46words extracted were used in the context including the contents of generic skills.

#### 3.1.2 Correspondence analysis by school

**Fig. 1:**
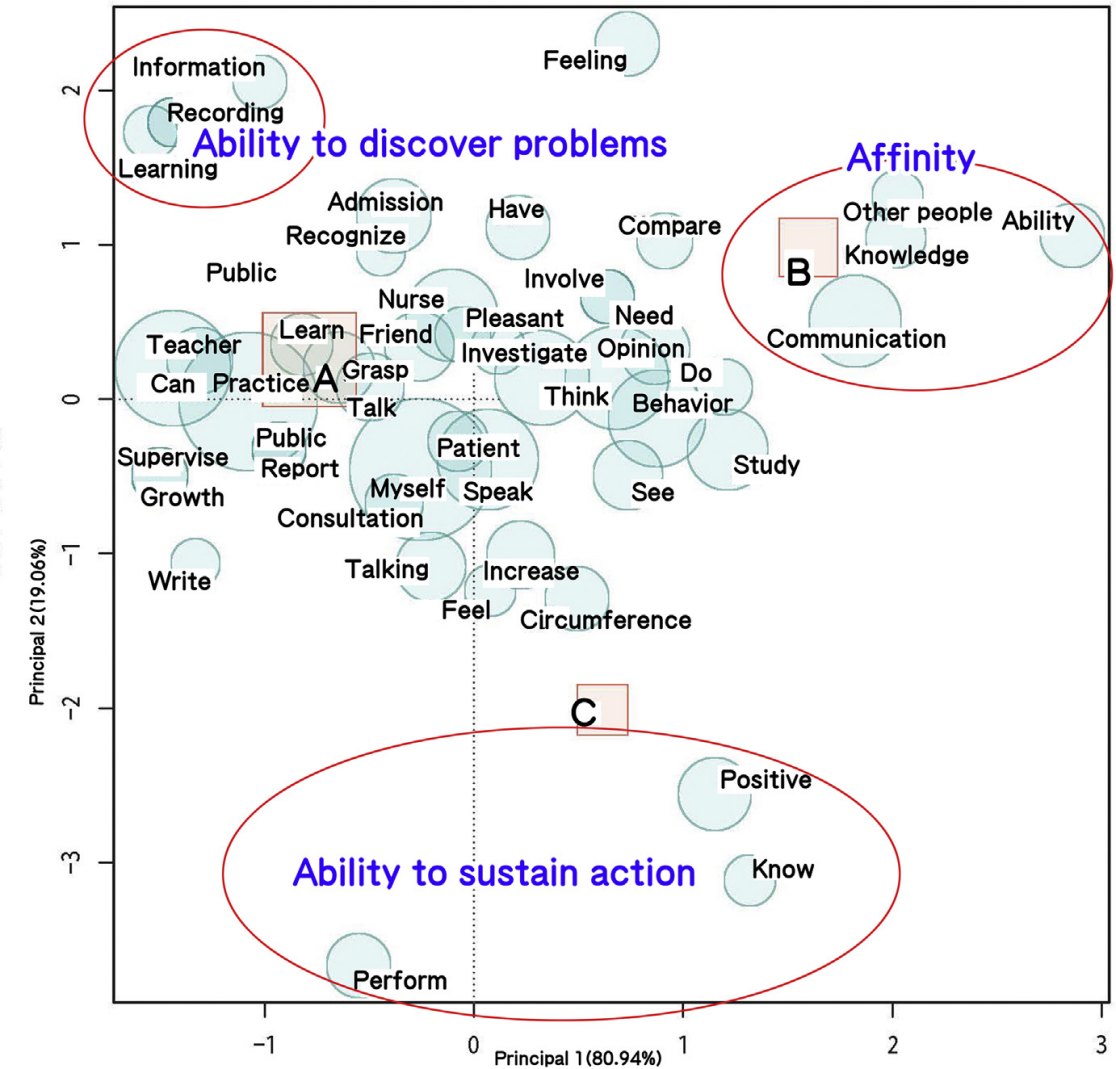
Correspondence analysis of open-ended questions. In the correspondence analysis, the higher the correlation between a word and another word or attribute, the closer it is plotted. In the overall placement of extracted words, on the positive side of the first quadrant, which is heavily populated, we find “communication/knowledge/other people/ability,” all words associated with the generic skill of “affinity.” In the second quadrant, we find “information/records/learning,” indicating “ability to discover problems” from the content of information gathering and analytical skills. Extracted terms in the second and third quadrants on the negative side of the second component are “proactive/know/perform,” all of which are included in “ability to sustain action.”

In the overall placement of extracted words, on the positive side of the first quadrant, which is heavily populated, we find “communication/knowledge/other people/ability,” all words associated with the generic skill of “affinity” (Fig. 1). “Affinity” is defined as “the ability to build harmonious interpersonal relationships” and consists of elements such as openness to others, tact, and interpersonal interest. Checking against the original text reveals passages such as the following: “Practical training has resulted in more smiling faces and better communication skills,” “Conversation with people who I meet for the first time is no longer an ordeal; I have a stronger desire to get to know the other person,” and “I am now able to offer my own opinion after having thought about the feelings of the other party.”

In the second quadrant, we find “information/records/learning,” indicating “ability to discover problems” from the content of information gathering and analytical skills. The “ability to discover problems,” which includes elements such as information gathering and causal analysis, indicates “the ability to clarify where problems lie and perform the requisite informational analysis.” In the original text from which the terms “information/records/learning” were extracted, the observed content included passage such as “I learned to gather the necessary information,” “I was able to organize information by keeping records,” and “By keeping lasting records (i.e., reflection sheets, assessment sheets, and evaluations and revisions of nursing plans), it became natural for me to think about nursing and assess my current self in terms of whether I am doing well as a human being.”

Extracted terms in the second and third quadrants on the negative side of the second component are “proactive/know/perform,” all of which are included in “ability to sustain action.” This is defined as “the ability to self-motivate and learn good habits,” which include learning behavior. These were found in descriptive passages such as “Although I had been passive, I became a little more proactive,” “I learned to be more proactive in my actions that before, the number of people I could speak with increased, and I was able to build cooperative relationships,” and “I started to be able to approach my instructors proactively and act on my own initiative.”

In terms of associations between phrases and attributes, associations were seen between “ability to discover problems” and School A, between “affinity” and School B, and “ability to sustain action” and School C.

#### 3.1.3 Analysis of characteristic words by school

**Fig. 2:**
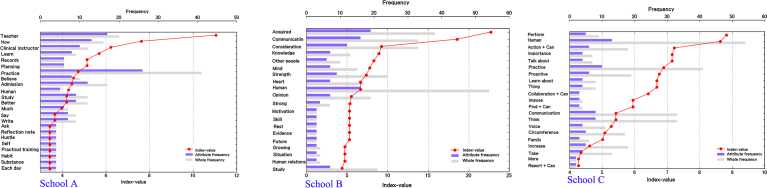
Characteristic word of each school. In characteristic word analysis, the objects of analysis were separated by school, whereupon characteristic words were extracted based on consideration of the frequencies of individual words based on the degree of complementary similarity as an index. Characteristic words are terms whose appearance is measured not in terms of simple frequency, but according to their bias toward certain attributes after the distribution of terms is considered. Growing Point - School A: We found “teacher,” “now,” and “clinical instructor” among the characteristic words for Attribute A, as well as “learn,” “record,” and “planning.” Growing Point - School B: The terms extracted for attribute B included “acquired,” “communication,” and “consideration.” Growing point - School C: Terms extracted for Attribute C included “perform,” “human,” and “action + can.”

To analyze differences by school, we extracted words that characteristically appeared in their respective groups (Fig. 2).

We found “teacher,” “now,” and “clinical instructor” among the characteristic words for School A, as well as “learn,” “records,” and “planning.” Checking the last two of these extracted terms against the original text, we find statements such as “I started planning everything out in my head before taking action” and “By keeping a record, I started to be able to clarify my next steps and I learned to be able to put everything together.”

The terms extracted for School B included “acquired,” “communication,” and “consideration.” In the text, these were found in statement such as “I acquired communication skills” and “I acquired the habit of consideration,” as well as “I acquired patience” and “I acquired the skill of putting things together.”

Terms extracted for School C included “perform,” “human,” and “action + can.” Three other terms were extracted in connection with the modal verb “can.”

## 4 Discussion

### 4.1 Associations between generic skills and areas of growth recognized by students

From the patterns in which the extracted words appeared, we were able to classify details reported by students regarding the areas where they recognized personal growth into nine categories, with each cluster including content associated with a generic skill. Although much debate has arisen over the idea of generic skills and their constituent elements, the clusters extracted in this case can be explained as constituent elements of generic skill competencies as defined by the Progress Report on Generic Skills, which is an assessment program jointly developed by the Kawaijuku Educational Institution and RIASEC, Inc., and supports the growth of generic skills (Andou et al., 2008).

The results of the present survey suggest that the personal growth recognized by students themselves in the context of clinical training was substantively related to generic skill competencies. Here, the term “competencies” refers to learned decision-making characteristics and behavioral styles for responding to surrounding situations. This corresponds to two key competencies outlined by the OECD's DeSeCo Project, namely, “the ability to interact in socially heterogeneous groups” and “the ability to act autonomously.” The former includes empathy, emotional control, and the ability to cooperate, while the latter includes elements such as reflection and objective self-evaluation (Kawaijuku Educational Institution and RIASEC, Inc., 2015).

Clinical training in the context of basic nursing education provides a setting for students to integrate the knowledge, techniques, and attitudes they have learned in lectures and exercises in assisting their patients (Andou et al., 2008; Maeda et al., 2000). It is also a setting in which students acquire practical nursing skills and problem-solving abilities through their experiences of putting nursing assistance into practice. However, students themselves perceived more growth in terms of generic skill competencies than in terms of practical nursing skills and specialized knowledge and techniques.

### 4.2 Associations between generic skills and practical training methods

The relationship between practical training methods and growth as recognized by students was considered based on the results of characteristic word analysis and the placement of extracted terms in the correspondence analysis.

“Ability to discover problems,” as the attribute most strongly associated with School A, is a skill that involves clarifying the location of problems and performing the requisite information analysis. The project-based learning practiced at School A begins by clarifying the goals of practical training, occasionally checking these goals during the training using a portfolio. A characteristic of this practice is that patients and personal tasks are constantly being clarified alongside goals (Suzuki, 2010). Also, from the use of the extracted key terms “records” and “planning” in the original texts, it seems that students perceived the “ability to discover problems” as personal growth in the repetition of this process. Moreover, as this content is consistent with the training goals adopted by School A, it may be considered the result of a learning effect.

In recent years, reports have discussed the use of portfolios in nursing education (Sakajo et al., 2013; Park and Byun, 2014; Choi and Kim, 2016). Although these are introduced in lectures and exercises, their effects can also be obtained via introduction in practical training, as seen from the results for School A in the present study. Using records in which students carry out repeated daily reflection, as in School A, can help to clarify personal challenges. In the course of learning, this allows students to build a foundation upon which to practice systematic self-reflection.

In clinical training, although students acquire concrete experience and then proceed to abstract conceptualization and active attempts at implementation, introspective reflection is positioned as a key step in this process (Waguri, 2010). It is suggested that records and engagements that elicit students' personal experiences and encourage reflection are effective for cultivating the “ability to discover problems” as a generic skill.

At School B, which shows a strong association with “affinity,” personal growth in the context of communication skills and the ability to form interpersonal relationships was also recognized from the results of characteristic word analysis. At School B, in the context of practical nursing training, the organization and analysis of information about patients' health problems and the nursing process, which consists of the implementation and evaluation of nursing plans, are being developed based on nursing theory. In this method, nursing practice toward patients constitutes the focus of evaluation. Moreover, the practical training methods at the center of the development of the nursing process are those that have been adopted by nursing training institutions in Japan. However, practical nursing training that emphasizes the nursing process is also marked by the problem that “specific patient elements are overemphasized, while the overall integrity and relationships of nursing are lost (Akutagawa and Katsuyama, 2007).” The results of the present study also indicate students themselves felt personal more growth in communication ability than in professional skills in the context of the nursing process and nursing practice. We may interpret this as a difference between the goals of the practical training method and students' own perceptions of growth.

“Ability to sustain action,” which was associated with School C, is a skill that entails taking initiative and acquiring good behaviors as habits. Clinical nursing training in other countries involves a method whereby hospital-based practice and lectures are repeated on a weekly basis, the lectures are used for learning about the cases and patient problems encountered in the practical component. However, because the Japanese nursing education system carries out clinical training in a specialized fashion, opportunities to make sense of practice are scarce. In view of this problem, Yasukata proposed the experiential practical training and education in 1997.

This method is based on John Dewey's pedagogical theory of “direct experience and reflective experience (Dewey, 1916).” This is a method that assists the process by which student learn from direct experience while undergoing repeated reflective experiences (Yasukata, 1999). Namely, she focuses on learning by acting, observing, and participating in the practice community. In experience-based practical training, students can objectively grasp the meaning of their own thoughts and actions by reflecting together with their instructors on the content of their experiences. Students' utilization of this understanding leads to more active behavior. In the characteristic word analysis as well, many terms were extracted featuring the modal verb “can,” which may indicate the effectiveness of experience-based practical training as a method that aims to cultivate proactive learning. The ability to learn proactively corresponds to the generic skill of “ability to sustain action,” and we find that experiential practical training also has an impact on generic skills.

The foregoing discussion suggests that clinical training has an impact on “ability to discover problems,” “affinity,” and “ability to sustain action,” and that this is associated with the methods used for practical training. Among these, the aims of the practical training methods used at School A and School C are consistent with the substance of students' growth. At these two schools, emphasis is placed on the process by which students verbalize introspective reflection and share and evaluate this together with their instructors. While it is clear that active learning and project-based learning lead to changes in generic skills (Yasuda et al., 2016), the key to this transformation seems to be the process of verbalizing introspective reflection.

### 4.3 Future outlook and strengths and limitations of this study

This study has examined the effect of clinical training on generic skills from the perspective of students' perceived self-growth. Because clinical training in nursing education is carried out based on the number of credits and the educational content set out in the Regulations of the Educational Institutions of Public Health Nurses, Midwives, and Nurses, clinical training was conceived of and implemented as a common intervention. However, since different schools were targeted, as well as differences in practical training methods, consideration must also be given to differences in other areas, such as the content of staff instruction, practical training instructors, and training settings.

On the other hand, the text mining analysis used in this study involved a quantitative analysis that made use of data mining methods to process and organize unstructured textual data in accordance with consistent rules by extracting useful information from large volumes of text (documents) (Saito, 2011). This enabled the analysis of large amounts of descriptive data, which had until now been a difficult proposition. The fact that text mining deals with textual information means that it can be applied to a variety of fields. In nursing, it can be used as a mixture of qualitative and quantitative methods. In the future, we believe there to be an inherent potential for development that will be able to obtain further findings by continuously carrying out future studies with an expanded range of target schools.

## 5 Conclusions

1. Students perceive more growth in terms of generic skill competencies than in terms of expert knowledge or techniques.
2. Project-based learning is associated with students' “ability to discover problems,” while experience-based practical training is associated with students' “ability to sustain action.”
3. In practical nursing training, the process of verbalizing student reflections and sharing and evaluating them together with teaching staff will bring about changes in generic skills.

## Declarations

### Author contribution statement

Hyeyong Lee: Conceived and designed the experiments; Performed the experiments; Analyzed and interpreted the data; Contributed reagents, materials, analysis tools or data; Wrote the paper.

Rie Shimotakahara: Performed the experiments; Analyzed and interpreted the data.

Akimi Fukada, Sumiko Shinbashi: Analyzed and interpreted the data; Contributed reagents, materials, analysis tools or data.

Shigemitsu Ogata: Conceived and designed the experiments; Wrote the paper.

### Funding statement

This work was supported by Japan Society for the Promotion of Science (KAKENHI:Grant-in-Aid for Young Scientists B # JP17K 17403).

### Competing interest statement

The authors declare no conflict of interest.

### Additional information

No additional information is available for this paper.
